# The relationship between distorted body image and lifestyle among Japanese adolescents: a population-based study

**DOI:** 10.1186/s13690-015-0082-z

**Published:** 2015-07-20

**Authors:** Takako Shirasawa, Hirotaka Ochiai, Hinako Nanri, Rimei Nishimura, Tadahiro Ohtsu, Hiromi Hoshino, Naoko Tajima, Akatsuki Kokaze

**Affiliations:** Department of Public Health, Showa University School of Medicine, Tokyo, Japan; Division of Diabetes, Metabolism and Endocrinology, Department of Internal Medicine, Jikei University School of Medicine, Tokyo, Japan; Jikei University School of Medicine, Tokyo, Japan

**Keywords:** Distorted body image, Lifestyle, Adolescent, Sex, Japanese

## Abstract

**Background:**

Distorted body image plays a significant role in the development of obesity, eating problems, and eating disorders. The aim of this study was to investigate the relationship between distorted body image and lifestyle among Japanese adolescent boys and girls.

**Methods:**

Subjects were 1731 seventh graders (age 12–13 years) from the Ina-town’s junior high schools, Japan, from 2005–2009. The height and weight of each subject were measured. Childhood underweight, overweight, and obesity were defined using the body mass index cutoff points proposed by the International Obesity Task Force. Information regarding the self-perceived weight status and lifestyles (exercise, snacking after dinner, breakfast, wakeup time, bedtime) of each subject was collected using a self-administered questionnaire. Self-perceived weight status was categorized into three groups (thin, normal, or heavy), and compared with the subjects’ actual weight status. Body image perception was categorized into the following three groups: an underestimated own weight status group (underestimated group), a correct own weight status group (correct group) and an overestimated own weight status group (overestimated group).

**Results:**

The proportion of boys in the underestimated group was higher than that of girls, while the opposite was true for the overestimated group (*P* < 0.001). There were no statistically significant differences in lifestyle between the underestimated group and the correct group regardless of sex. In contrast, there were statistically significant differences between the overestimated group and the correct group in the lifestyle factors of exercise among boys and snacking after dinner among girls. The adjusted odds ratio (OR) in boys who exercised daily significantly decreased (OR: 0.35, 95 % CI: 0.16–0.77), while a significantly increased OR was observed in girls who snacked after dinner (OR: 1.53, 95 % CI: 1.07–2.19).

**Conclusion:**

Adolescent boys tended to underestimate their body weight, whereas adolescent girls were likely to overestimate their body weight. Furthermore, lifestyle factors associated with distorted body image differed by sex, with exercise affecting body image perception among boys and snacking after dinner affecting body image perception among girls. Thus, lifestyle may lead to distorted body image among adolescents.

## Background

Childhood overweight and obesity have increased dramatically in economically developed countries and in urbanized populations [1]. In Japan, Matsushita et al. showed increasing trends in obesity prevalence in school children [2]. However, Inokuchi et al. reported that the prevalence of thinness among Japanese adolescents has progressively increased [3, 4]. Because thinness, overweight and obesity in adolescence have several adverse effects on health [5, 6], these are serious public health issues.

Body image plays a significant role in the development of obesity [7], eating problems, and eating disorders [8]. Some studies have reported that distorted body image was an important factor related to abnormal eating attitudes and behaviors among young people [9–11]. Kiriike et al. showed that the body mass index (BMI) of adolescents in Japan has decreased and that body shape has become thinner as a result of dieting in order to become slim [12]. Therefore, body image is an important factor for healthy body weight.

The relationship between body image and lifestyle factors, such as physical activity and eating behavior, among adolescents has been reported from many countries [13–18]. To the best of our knowledge, only one study examined the relationship between a distorted perception of body image and lifestyle factors (breakfast, snack, night snack, eating speed, eating amount, wakeup time, bedtime, exercise) in Japanese adolescent girls [19], while there have been no studies on this association among adolescent boys. Because body image is reported to be a strongly gendered phenomenon [20], it is necessary to investigate the relationship between distorted body image and lifestyle for each sex among adolescents.

Accordingly, the aim of this study was to investigate the relationship between distorted body image and lifestyle for each sex in Japanese adolescents.

## Methods

### Subjects

As a part of its community health services, the town of Ina in Saitama Prefecture, Japan has provided a unique health promotion program to prevent childhood lifestyle-related diseases since 1994, in addition to the annual national health checkups performed in accordance with the School Health Law of Japan. The program consists of a questionnaire survey and physical examinations for fourth and seventh graders. The present study was conducted as a part of this program. The study subjects comprised all seventh graders (age 12–13 years) from Ina-town’s junior high schools from 2005 to 2009 (*N* = 1821). Written informed consent was obtained from each child’s parent or guardian. The study protocol was approved by the Medical Ethics Committee of Showa University School of Medicine.

### Anthropometric measurements

Measurements of height and weight for each child were performed annually during 2005–2009. The same examination protocol was used annually to ensure uniformity of quality and precision of assessment. For the measurements, all children were asked to remove their shoes and socks, after which their height and weight were measured in increments of 0.1 cm and 0.1 kg, respectively, while they were wearing light clothing. BMI was calculated as body weight (kg) divided by the square of the height (m^2^). Childhood underweight, overweight, and obesity were defined using the BMI cutoff points (i.e., the age- and sex-specific cutoff points that were linked to BMI values of 18.5, 25 and 30 at age 18, respectively) proposed by the International Obesity Task Force [21–23]. The BMI cut-off points were based on averaging data from six countries including Asian countries, and the cut-off points were shown to be applicable to Japanese children [24]. In the present study, the BMI cut-off points for childhood underweight, overweight, and obesity were 15.35, 21.22, and 26.02 (age 12) and 15.84, 21.91, and 26.84 (age 13) for boys, and 15.62, 21.68, and 26.67 (age 12) and 16.26, 22.58, and 27.76 (age 13) for girls, respectively. In the present study, obesity was included in overweight. Children who were not underweight or overweight were regarded as normal weight. Therefore, each subject in this study was categorized into one of three actual weight status categories (underweight, normal weight, or overweight).

### Body image perception

Self-perceived weight status was assessed using the following question on a self-administered questionnaire given to each child: “Do you think you are very thin, thin, normal weight, heavy, or very heavy?” Self-perceived weight status was then categorized into three groups: thin (“very thin” and “thin”), normal (“normal weight”) and heavy (“heavy” and “very heavy”). Based on a comparison between the self-perceived weight status (thin, normal, or heavy) of each subject and his or her actual weight status (underweight, normal, or overweight), body image perception was categorized into the following three groups: an underestimated own weight status group (underestimated group), a correct own weight status group (correct group) and an overestimated own weight status group (overestimated group). For instance, subjects with a normal weight who perceived themselves as thin were included in the underestimated group, while those who perceived themselves as heavy were included in the overestimated group. Underestimated weight or overestimated weight were regarded as distorted body images in the present study.

### Lifestyle factors

Information on the following lifestyle factors was obtained using a self-reported questionnaire given to each subject: snacking after dinner, and exercise other than physical education class. Each subject’s parent or guardian was asked to complete a self-administered questionnaire regarding the breakfast, wakeup time and bedtime of each subject. Sleeping hours were calculated from wakeup time and bedtime.

### Statistical analysis

The unpaired *t*-test or chi-squared test was used to compare characteristics between boys and girls. In the analysis stratified by sex, lifestyle factors in the correct group were compared with those in the underestimated and overestimated groups. Adjusted odds ratios (ORs) and 95 % confidence intervals (CIs) were calculated by logistic regression. A *P* value of less than 0.05 was considered statistically significant. All data were analyzed using SPSS 20.0J (IBM, Chicago, IL, USA).

## Results

Among the 1821 children, 21 refused to participate in the program (participation rate: 98.8 %), and 69 were excluded due to missing data on anthropometric measurements and self-perceived weight status. Thus, data from a total of 1731 children (885 boys and 846 girls) were analyzed. The characteristics of the subjects are shown in Table 1. There was no statistically significant difference between boys and girls in actual weight status (underweight, normal weight, overweight) (*P* = 0.116). Self-perceived weight status and body image perception were statistically significantly different between boys and girls, respectively (*P* < 0.001). The proportion of boys in the underestimated group was higher than that of girls, whereas the proportion of girls in the overestimated group was higher than that of boys (*P* < 0.001). About 70 % of the children perceived their actual weight status correctly regardless of sex.

**Table 1:** Characteristics of study participants by sex (Japan; 2005–2009)

Variable	Boys (*n* = 885)	Girls (*n* = 846)	*P*-value
Age (years)	12.3 (0.4)	12.3 (0.4)	0.631
Height (cm)	154.4 (8.1)	152.5 (6.0)	<0.001
Weight (kg)	44.5 (9.7)	43.6 (7.9)	0.040
Body mass index (kg/m^2^)	18.5 (3.0)	18.7 (2.7)	0.276
Actual weight (%)			
Underweight	73 (8.2)	88 (10.4)	0.116
Normal weight	694 (78.4)	666 (78.7)	
Overweight	118 (13.3)	92 (10.9)	
Self-perceived weight status (%)			
Thin	268 (30.3)	139 (16.4)	<0.001
Normal	484 (54.7)	506 (59.8)	
Heavy	133 (15.0)	201 (23.8)	
Body image perception (%)			
Underestimated	230 (26.0)	99 (11.7)	<0.001
Correct	605 (68.4)	591 (69.9)	
Overestimated	50 (5.6)	156 (18.4)	

Data are expressed as numbers (%), values are means (standard deviation)

The unpaired *t*-test and chi-squared test were used to compare characteristics between boys and girls

**Fig. 1:**
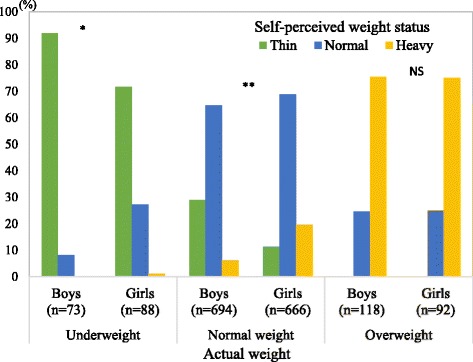
Actual weight and self-perceived weight status by sex (Japan; 2005–2009). Chi-square test for analysing sex differences (NS: not significant; *P < 0.05; **P < 0.01)

Figure 1 shows the relationship between actual weight status and self-perceived weight status by sex. Over 90 % of underweight boys perceived themselves as thin, whereas about 30 % of underweight girls perceived themselves as normal or heavy. In addition, approximately 30 % of normal weight boys underestimated their actual weight status. In contrast, about 20 % of normal weight girls overestimated their actual weight status. Among overweight children, over 20 % underestimated their actual weight status regardless of sex.

The relationship between lifestyle and body image perception by sex are shown in Tables 2, 3, 4 and 5. There were no statistically significant differences in lifestyle between the underestimated group and the correct group regardless of sex (Tables 2 and 3). In contrast, there were statistically significant differences between the overestimated group and the correct group in the lifestyle factors of exercise among boys (Table 4) and snacking after dinner among girls (Table 5). The adjusted odds ratio (OR) in boys who exercised daily significantly decreased (OR: 0.35, 95 % CI: 0.16–0.77), while a significantly increased OR was observed in girls who snacked after dinner (OR: 1.53, 95 % CI: 1.07–2.19). There were no statistically significant associations between overestimated body weight image and wakeup time, bedtime, or sleeping hours in either sex.

**Table 2:** Relationship between lifestyle factors and distorted body image (underestimation) among boys (Japan; 2005–2009)

	Body image perception, n (%)		Adjusted^a^ OR (95 % CI)	*P*-value
	Correct	Underestimated		
Exercise				
Daily	507 (86.7)	188 (85.8)	0.85 (0.46–1.57)	0.609
Sometimes	35 (6.0)	14 (6.4)	0.84 (0.36–2.00)	0.699
Never	43 (7.4)	17 (7.8)	1.00	
Snacking after dinner				
Yes	314 (52.7)	121 (54.0)	1.05 (0.76–1.44)	0.774
No	282 (47.3)	104 (46.0)	1.00	
Skipping breakfast				
No	568 (95.5)	216 (95.2)	1.00	
Yes	27 (4.5)	11 (4.8)	1.10 (0.53–2.29)	0.795
Wakeup time				
Before 6:30	298 (52.6)	111 (49.6)	1.00	
6:30–6:59	84 (14.8)	34 (15.2)	1.24 (0.77–1.98)	0.374
After 7:00	185 (32.6)	79 (35.3)	1.20 (0.84–1.70)	0.319
Bedtime				
Before 22:00	58 (10.6)	24 (11.0)	1.00	
22:00–22:59	298 (54.7)	119 (54.6)	1.00 (0.59–1.70)	1.000
After 23:00	189 (34.7)	75 (34.4)	1.05 (0.60–1.83)	0.867
Hours of sleep				
< 8	110 (20.6)	44 (20.4)	1.00	
8–9	302 (56.7)	120 (55.6)	0.92 (0.61–1.40)	0.705
9+	121 (22.7)	52 (24.1)	0.99 (0.61–1.61)	0.964

OR, odds ratio; 95 % CI, 95 % confidence interval

^a^Age and body mass index were adjusted in a logistic regression analysis

**Table 3:** Relationship between lifestyle factors and distorted body image (underestimation) among girls (Japan; 2005–2009)

	Body image perception, n (%)		Adjusted^a^ OR (95 % CI)	*P*-value
	Correct	Underestimated		
Exercise				
Daily	343 (60.0)	63 (65.6)	1.28 (0.76–2.16)	0.346
Sometimes	68 (11.9)	10 (10.4)	1.05 (0.47–2.35)	0.903
Never	161 (28.1)	23 (24.0)	1.00	
Snacking after dinner				
Yes	276 (47.8)	56 (57.1)	1.44 (0.93–2.23)	0.100
No	302 (52.2)	42 (42.9)	1.00	
Skipping breakfast				
No	548 (93.4)	91 (91.9)	1.00	
Yes	39 (6.6)	8 (8.1)	1.33 (0.60–2.98)	0.485
Wakeup time				
Before 6:30	318 (55.8)	45 (46.4)	1.00	
6:30–6:59	90 (15.8)	17 (17.5)	1.36 (0.74–2.51)	0.329
After 7:00	162 (28.4)	35 (36.1)	1.62 (1.00–2.64)	0.052
Bedtime				
Before 22:00	36 (6.7)	9 (9.5)	1.00	
22:00–22:59	262 (48.4)	46 (48.4)	0.73 (0.33–1.63)	0.445
After 23:00	243 (44.9)	40 (42.1)	0.72 (0.32–1.63)	0.435
Hours of sleep				
< 8	176 (32.8)	22 (23.4)	1.00	
8–9	288 (53.7)	54 (57.4)	1.44 (0.85–2.46)	0.179
9+	72 (13.4)	18 (19.1)	1.85 (0.93–3.67)	0.081

OR, odds ratio; 95 % CI, 95 % confidence interval

^a^Age and body mass index were adjusted in a logistic regression analysis

**Table 4:** Relationship between lifestyle factors and distorted body image (overestimation) among boys (Japan; 2005–2009)

	Body image perception, n (%)		Adjusted^a^ OR (95 % CI)	*P*-value
	Correct	Overestimated		
Exercise				
Daily	507 (86.7)	37 (75.5)	0.35 (0.16–0.77)	0.009
Sometimes	35 (6.0)	3 (6.1)	0.41 (0.10–1.65)	0.210
Never	43 (7.4)	9 (18.4)	1.00	
Snacking after dinner				
Yes	314 (52.7)	22 (44.0)	0.69 (0.39–1.24)	0.219
No	282 (47.3)	28 (56.0)	1.00	
Skipping breakfast				
No	568 (95.5)	45 (91.8)	1.00	
Yes	27 (4.5)	4 (8.2)	1.87 (0.63–5.59)	0.262
Wakeup time				
Before 6:30	298 (52.6)	23 (46.9)	1.00	
6:30–6:59	84 (14.8)	7 (14.3)	1.09 (0.45–2.65)	0.842
After 7:00	185 (32.6)	19 (38.8)	1.31 (0.69–2.48)	0.403
Bedtime				
Before 22:00	58 (10.6)	3 (6.8)	1.00	
22:00–22:59	298 (54.7)	26 (59.1)	1.63 (0.48–5.59)	0.434
After 23:00	189 (34.7)	15 (34.1)	1.46 (0.41–5.25)	0.561
Hours of sleep				
< 8	110 (20.6)	7 (15.9)	1.00	
8–9	302 (56.7)	28 (63.6)	1.45 (0.61–3.42)	0.397
9+	121 (22.7)	9 (20.5)	1.20 (0.43–3.35)	0.722

OR odds ratio, 95 % CI 95 % confidence interval

^a^Age and body mass index were adjusted in a logistic regression analysis

**Table 5:** Relationship between lifestyle factors and distorted body image (overestimation) among girls (Japan; 2005–2009)

	Body image perception, n(%)		Adjusted^a^ OR (95 % CI)	*P*-value
	Correct	Overestimated		
Exercise				
Daily	343 (60.0)	81 (52.9)	0.87 (0.58–1.32)	0.523
Sometimes	68 (11.9)	28 (18.3)	1.51 (0.87–2.62)	0.147
Never	161 (28.1)	44 (28.8)	1.00	
Snacking after dinner				
Yes	276 (47.8)	90 (58.4)	1.53 (1.07–2.19)	0.021
No	302 (52.2)	64 (41.6)	1.00	
Skipping breakfast				
No	548 (93.4)	147 (95.5)	1.00	
Yes	39 (6.6)	7 (4.5)	0.67 (0.30–1.54)	0.347
Wakeup time				
Before 6:30	318 (55.8)	89 (59.7)	1.00	
6:30–6:59	90 (15.8)	25 (16.8)	0.98 (0.60–1.63)	0.950
After 7:00	162 (28.4)	35 (23.5)	0.76 (0.49–1.17)	0.215
Bedtime				
Before 22:00	36 (6.7)	8 (5.8)	1.00	
22:00–22:59	262 (48.4)	66 (47.8)	1.12 (0.50–2.54)	0.780
After 23:00	243 (44.9)	64 (46.4)	1.15 (0.51–2.60)	0.738
Hours of sleep				
< 8	176 (32.8)	49 (35.8)	1.00	
8–9	288 (53.7)	72 (52.6)	0.92 (0.61–1.38)	0.672
9+	72 (13.4)	16 (11.7)	0.82 (0.44–1.55)	0.544

OR, odds ratio; 95 % CI, 95 % confidence interval

^a^Age and body mass index were adjusted in a logistic regression analysis

## Discussion

The present study demonstrated that about 70 % of children evaluated their actual weight status appropriately regardless of sex. It follows from this that 30 % of adolescent boys and girls overestimated or underestimated their actual weight status. In addition, adolescent boys tended to underestimate their body weight, whereas adolescent girls were more likely to overestimate their body weight, findings consistent with previous results [25–27]. In a previous study, the desire for thinness and fear of weight gain were shown in significant proportions of preadolescent girls and progressively increased to include the majority of adolescent girls [28]. Among girls, the preference for an extremely slim body might have roots in the lack of proper understanding of average body weight, which might also be affected by the fact that they set their ideal shape at a level that is even lower than their misunderstood average [29]. In contrast to the girls who want to be slim, adolescent boys might be increasingly concerned with becoming more muscular [20]. Therefore, sex differences in body image perception could be due to the difference in body preference between boys and girls.

In this study, no significant relationship between distorted body image (underestimated weight) and lifestyle was observed among either sex. The results suggested that the lifestyle of adolescents in the underestimated group was similar to that of adolescents in the correct group regardless of sex. However, because adolescents in the underestimated group did not estimate their actual body weight accurately, their distorted body image might have adverse effects on their future health.

In contrast, distorted body image (overestimated weight) was significantly associated with some lifestyle factors. Although previous studies have reported the association between lifestyle factors (diet, physical activity habits and sedentary behaviors) and overweight/obesity among adolescents [24, 30, 31], these lifestyle factors were related to distorted body image independently of actual BMI in this study. Moreover, the impact of lifestyle on overestimating body weight varied by sex; overestimated body weight was positively associated with snacking after dinner among girls, while it was negatively associated with exercise behavior among boys. Boys with regular exercise habits might not tend to overestimate their body weight, which may have been the reason for the low OR for overestimated body weight in this study. Adolescent girls who overestimated their body weight tended to snack after dinner, a finding consistent with a previous study [32]. Among adolescent girls, eating faster and not eating breakfast on a daily basis were identified as being associated with overestimated body weight [19]. These findings suggest that the presence of such unhealthy behaviors during adolescence might have long-term detrimental effects on body image in both sexes.

Most studies about body image among Japanese adolescents have used self-reported weight and height to calculate BMI, which has been reported to be inaccurate [8]. In contrast, the height and weight of over 1800 population-based schoolchildren were measured at a high participation rate (over 98 %) in our study, which was its main strength. However, the present study also had some limitations. First, this study did not consider socio-economic status [32] and psychological factors [33], which have been shown to influence weight perception. Second, the subjects in this study were seventh graders (12–13 years of age) from one town in Japan, which might limit generalizability to other Japanese schoolchildren or other races. Finally, the present study could not determine a causal relationship between a distorted body image and lifestyle since this was a cross-sectional study. Therefore, the possibility of reverse causality cannot be denied.

## Conclusions

The present study found that adolescent boys tended to underestimate their body weight, whereas adolescent girls were likely to overestimate their body weight. Furthermore, lifestyle factors associated with a distorted body image differed by sex, with exercise affecting perception among boys and snacking after dinner affecting perception among girls. Thus, lifestyle may lead to distorted body image among adolescents. Therefore, sex differences should be considered when educating adolescents on accurate weight perception, which could contribute to public health strategies to curb the increasing prevalence of underweight and overweight/obesity in Japan.
